# OP08 Health Technology Assessments Of Artificial Intelligence: Special Considerations And Development Of A Checklist

**DOI:** 10.1017/S0266462324000722

**Published:** 2025-01-07

**Authors:** Leona Batten, Antonia Needham-Taylor, Clare England, David Jarrom

## Abstract

**Introduction:**

Artificial intelligence (AI) technologies are becoming more widespread in daily life, including in health and care settings. AI is being developed for use in many areas, including diagnostics, treatment planning, and patient monitoring. There are new challenges and additional considerations when performing a health technology assessment (HTA) for AI use in healthcare.

**Methods:**

Health Technology Wales has developed additional guidance and a checklist of considerations for researchers to use when undertaking an HTA on AI. These documents were created as a result of involvement in discussions with healthcare services around AI; attendance at meetings and training sessions with government, healthcare, and academic institutions; and observations from undertaking our first HTAs investigating AI technologies.

**Results:**

We identified seven main areas to consider: (i) the AI model being used and its training datasets; (ii) clarity on where in the clinical pathway the AI sits and whether this is appropriate for the local healthcare service; (iii) clear identification of the target patient population and whether there are gaps in the evidence base for subgroups; (iv) who uses the AI and the training required; (v) possible barriers and inequities in usage, particularly if the AI is patient facing; (vi) ongoing monitoring of the AI model; and (vii) data security and usability.

**Conclusions:**

HTA assessment of AI presents new challenges for HTA bodies. Additional outcomes for assessment (including user acceptability) may be required, and an awareness of background information that allows for contextualization of the technology and clear indication of its usefulness in the local healthcare setting is key. The development of guidance related to AI technologies may enable rigorous evaluation.

